# Increased Goal Tracking in Adolescent Rats Is Goal-Directed and Not Habit-Like

**DOI:** 10.3389/fnbeh.2019.00291

**Published:** 2020-01-14

**Authors:** Analise N. Rode, Bita Moghaddam, Sara E. Morrison

**Affiliations:** Department of Neuroscience, University of Pittsburgh, Pittsburgh, PA, United States

**Keywords:** adolescent, rat, sign tracking, goal tracking, habit, devaluation, Pavlovian conditioning

## Abstract

When a cue is paired with reward in a different location, some animals will approach the site of reward during the cue, a behavior called goal tracking, while other animals will approach and interact with the cue itself: a behavior called sign tracking. Sign tracking is thought to reflect a tendency to transfer incentive salience from the reward to the cue. Adolescence is a time of heightened sensitivity to rewards, including environmental cues that have been associated with rewards, which may account for increased impulsivity and vulnerability to drug abuse. Surprisingly, however, studies have shown that adolescents are actually less likely to interact with the cue (i.e., sign track) than adult animals. We reasoned that adolescents might show decreased sign tracking, accompanied by increased apparent goal tracking, because they tend to attribute incentive salience to a more reward-proximal “cue”: the food magazine. On the other hand, adolescence is also a time of enhanced exploratory behavior, novelty-seeking, and behavioral flexibility. Therefore, adolescents might truly express more goal-directed reward-seeking and less inflexible habit-like approach to a reward-associated cue. Using a reward devaluation procedure to distinguish between these two hypotheses, we found that adolescents indeed exhibit more goal tracking, and less sign tracking, than a comparable group of adults. Moreover, adolescents’ goal tracking behavior is highly sensitive to reward devaluation and therefore goal-directed and not habit-like.

## Introduction

Animals and humans vary widely in the degree to which they ascribe motivational value, or incentive salience, to reward-predictive cues. This variability can be measured using a Pavlovian conditioned approach (PCA) procedure in which a cue (e.g., extension of a lever) is followed by the delivery of a reward in a separate location. Under these circumstances, some animals will approach the location of reward delivery: a behavior known as goal tracking (Boakes, 1977). Other animals will approach and interact with the cue itself, a behavior known as sign tracking (Hearst and Jenkins, 1974). A growing body of evidence supports a relationship between sign tracking and certain maladaptive behaviors, including impulsive action (Lovic et al., 2011), initiation and maintenance of drug-taking (Flagel et al., 2009; Beckmann et al., 2011) and relapse after abstinence (Versaggi et al., 2016).

In both humans and non-human animals, adolescence is a time of enhanced sensitivity to rewards, including natural rewards such as sugar (Friemel et al., 2010) and drug rewards such as nicotine (Dannenhoffer and Spear, 2016) and alcohol (Doremus-Fitzwater and Spear, 2016). In many contexts, adolescents also exhibit greater behavioral and neural responsivity to cues that predict reward (Sturman et al., 2010; Burton et al., 2011; Sturman and Moghaddam, 2011). Finally, adolescents display enhanced impulsivity in tasks such as the 5-CSRTT (Burton and Fletcher, 2012). Given these factors, combined with heightened risk-taking (Gardner and Steinberg, 2005; Westbrook et al., 2018), it is unsurprising that adolescents are especially vulnerable to substance abuse (Chambers et al., 2003). Because sign tracking (ST) is also associated with factors contributing to drug abuse and addiction, it is reasonable to hypothesize that adolescents would be more prone to ST. However, studies have found the opposite: under normal circumstances, adolescents typically exhibit lower levels of sign tracking than adults (Anderson and Spear, 2011; Doremus-Fitzwater and Spear, 2011). Only under conditions of heightened stress, such as social isolation combined with food restriction, do adolescents develop equivalent or enhanced ST behavior relative to adults (Anderson et al., 2013; DeAngeli et al., 2017).

These observations give rise to two competing hypotheses. One possibility is that adolescents might display less apparent sign tracking than adults because they ascribe incentive salience to one or more alternative targets, such as the food magazine. This notion is supported by the finding that, among adult sign trackers, most apparent “goal tracking” behavior (which occurs alongside sign tracking in many animals) is insensitive to reward devaluation (Morrison et al., 2015). In other words, subjects that are predisposed to sign tracking in general may exhibit ST-like behavior directed toward the location of reward, not just the cue.

On the other hand, adolescence is a time of enhanced exploratory activity and novelty-seeking (Douglas et al., 2003), and adolescent rats often exhibit more cognitive and behavioral flexibility than adults (Simon et al., 2013; Westbrook et al., 2018). Indeed, some studies have shown that, despite their higher reward sensitivity, adolescents are less prone to habit formation than adults, including drug-seeking habits (Serlin and Torregrossa, 2015) and habits emerging from the pursuit of natural rewards (Naneix et al., 2012). Given these findings, we might expect to see more goal-directed behavior among adolescents (manifested as goal tracking) and less inflexible, habit-like conditioned approach towards a reward-associated cue (i.e., sign tracking).

To distinguish between these two hypotheses, we examined goal-directed vs. habit-like behavior in adolescents compared to adults in a Pavlovian setting. Adolescent and adult male rats were trained on a PCA task and subjected to a reward devaluation procedure to evaluate whether adolescents’ lever- and magazine-oriented behavior is primarily goal-directed or habit-like.

## Materials and Methods

All procedures were performed in accordance with the standards of the National Institutes of Health and were approved by the Institutional Animal Care and Use Committee of the University of Pittsburgh.

### Subjects

Subjects were 32 adolescent male Long-Evans rats obtained from Charles River Laboratory at 21 days of age. An adult comparison group consisted of 39 male Long-Evans rats obtained from Charles River Laboratory weighing 275–300 g upon arrival (approximately 9 weeks). All rats were allowed to acclimate to the housing colony for 7 days, and then habituated to gentle handling over the next 7 days. Among adolescents, training and experimental procedures took place between postnatal days 35 and 51. All animals were pair-housed on a 12 h light/dark cycle and all procedures took place during the dark period. Mild food restriction was initiated 2 days prior to the start of training, with adolescents receiving 10 g and adults 14 g of chow per day. Rats were weighed regularly to ensure that they did not fall below 85% of pre-restriction body weight (adults) or 85% of the weight of age-matched free-feeding controls (adolescents).

### Apparatus and Training

Training and experiments took place in a standard operant chamber (Coulbourn Instruments) controlled by GraphicState 3.0 and equipped with a house light, pellet delivery system, food magazine recessed into the side wall, and a single retractable lever to one side of the magazine (counterbalanced among subjects). A white cue light was located above the lever. The magazine was equipped with an infrared photo-detector to record entries and exits.

Rats were trained using a PCA procedure similar to that used previously (Gillis and Morrison, 2019). Subjects were initially trained to retrieve sugar pellets (45 mg, Bio-Serv) from the magazine over two sessions consisting of 50 pellets delivered individually on a variable interval schedule averaging 60 s. Rats then received seven daily acquisition sessions on the PCA task, consisting of 25 trials separated by an interval selected from a truncated exponential distribution averaging 60 s. Trials began with the presentation of the cue, consisting of lever extension and a flashing cue light (5 Hz) for 8 s. Upon completion of the cue, the lever retracted, the cue light was extinguished, and a sugar pellet was delivered to the magazine.

### Reward Devaluation and Testing

After 7 days of training on the PCA task, rats were subjected to devaluation of the sugar pellet reward *via* taste aversion conditioning. Rats were divided into behavior-matched groups based on the PCA index (see below) calculated for the last day of training. The two groups received either reward devaluation (“paired” group) or sham devaluation (“unpaired”). Rats in the paired group were given access to 50 sugar pellets over 10 min in an empty cage. Immediately afterward, both groups were injected with lithium chloride (LiCl; 0.6 M; 5 ml/kg i.p.). The next day, rats in the unpaired group were given similar access to sugar pellets. Immediately afterward, both groups were injected with vehicle (0.9% saline). Thus, both groups experienced the same exposure to sugar pellets and the same injections, but only the paired group experienced the sugar pellets in conjunction with lithium.

The day after vehicle injections, rats were given a test session in extinction. The test session was identical to the training sessions except that no rewards were delivered. On the same day, rats were given a consumption test consisting of 10 min of exposure to 50 sugar pellets in an empty cage.

### Data Analysis

We quantified sign tracking and goal tracking by calculating a PCA index (Meyer et al., 2012) for each individual, which comprises the average of three ratios: (1) probability index, which compares the probability of lever deflection and magazine entry during the cue, calculated as (P_lever_−P_magazine_); (2) bias index, which compares the average number of lever deflections and magazine entries per cue presentation, calculated as (#lever − #receptacle/#lever + #receptacle); and (3) latency index, which compares the average latency from cue onset to lever deflection vs. magazine entry, calculated as (lat_magazine_−lat_lever_)/(cue length). When a behavior was not performed, the latency was defined as the cue length. Each of these indices ranges from −1.0 to +1.0, where more positive numbers indicate more sign tracking (relative to goal tracking) and more negative numbers indicate more goal tracking (relative to sign tracking).

In order to isolate the effects of devaluation from the effects of extinction, analyses were performed on data from the first 10 trials of both training and test sessions. As in some previous reports (e.g., Tunstall and Kearns, 2015), we operationally defined “sign trackers” as animals with an average PCA index greater than zero and “goal trackers” as animals with an average PCA index less than zero. Although in previous studies (e.g., Morrison et al., 2015) we have categorized animals using the mean or median PCA index of a specific group of animals, here we opted to use a fixed boundary in order to facilitate comparisons across groups (adults and adolescents).

All statistical comparisons were performed using a Wilcoxon signed-rank test (within-group comparisons) or a Wilcoxon rank-sum test (across-group comparisons). Where appropriate, alphas were corrected for multiple comparisons using the Holm-Sidak method.

## Results

We trained 32 adolescents and 39 adult rats in a PCA procedure similar to those we have used previously to study sign tracking and goal tracking behavior in adults (Morrison et al., 2015; Gillis and Morrison, 2019). In this task, sign tracking is represented by lever deflections and goal tracking by magazine entries during the 8 s lever/light cue, although neither behavior is required for delivery of the sugar pellet reward after cue termination.

### Acquisition of Sign Tracking and Goal Tracking

**Figure 1:**
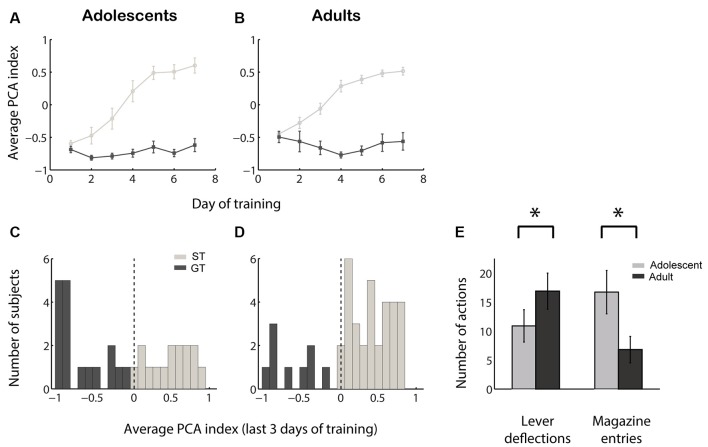
Adolescents perform less sign tracking and more goal tracking than adults. **(A,B)** Average Pavlovian conditioned approach (PCA) index among sign trackers (light gray) and goal trackers (dark gray) over 7 days of training for adolescents **(A)** and adults **(B)**. Higher PCA index indicates more sign tracking relative to goal tracking. **(C,D)** Average PCA index for adolescents **(C)** and adults **(D)** over the last 3 days of the 7-day training period. Individuals categorized as sign trackers (ST) and goal trackers (GT) are indicated in light gray and dark gray, respectively. **(E)** Average raw counts of lever deflections and magazine entries during the cue over the last 3 days of training for adolescents (gray) and adults (black). Error bars, SEM. Asterisk, *p* < 0.05.

We quantified the tendency of individuals towards sign-tracking vs. goal-tracking behavior by calculating a PCA index ranging from −1.0 (all GT, no ST) to +1.0 (all ST, no GT). The acquisition of sign tracking and goal tracking behaviors followed a similar time course among adolescent vs. adult sign trackers and goal trackers (Figures 1A,B). At the end of the 7 days of training, adolescent rats exhibited a slight but non-significant population bias towards goal tracking (Figure 1C; median PCA index not different from 0; *Z* = −1.12, *p* = 0.26) while adult rats exhibited a marked population bias towards sign tracking (Figure 1D; median PCA index >0, *Z* = 3.18, *p* = 0.002). The distribution of PCA index was significantly different between the two groups (*Z* = −2.60, *p* = 0.01), and this difference was driven by both fewer lever deflections (*Z* = −2.00, *p* = 0.046) and more magazine entries (*Z* = 2.88, *p* = 0.004) among adolescents compared to adults (Figure 1E). Thus, not only do adolescents perform fewer sign-tracking actions than adults, confirming prior findings (Anderson and Spear, 2011; Doremus-Fitzwater and Spear, 2011), they also perform more goal-tracking behavior.

### Effects of Reward Devaluation

After 7 days of training on the PCA task, adolescent rats underwent reward devaluation *via* taste aversion conditioning: subjects in the “paired” group were given sugar pellets outside of the task environment, followed immediately by injection with LiCl solution to induce illness. Subjects in the “unpaired” group were also injected with LiCl; then, on a subsequent day, the unpaired group was allowed to eat sugar pellets, followed immediately by vehicle injection for all rats. Thus, all rats experienced the same exposure to sugar pellets and injections, but only the paired group experienced the temporal conjunction of sugar pellets and illness.

**Figure 2:**
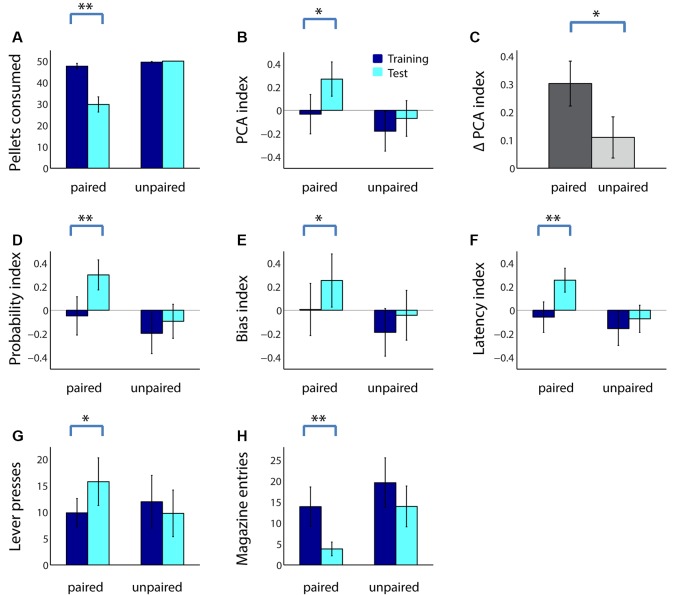
Reward devaluation increases sign tracking and decreases goal tracking in adolescent rats. **(A)** The average number of sugar pellets consumed before (dark blue) and after (cyan) reward devaluation (paired group) or sham devaluation (unpaired group). **(B)** Average PCA index over the last 3 days of training dark (blue) and in the test session (cyan) for the paired and unpaired groups. Higher PCA index indicates more sign tracking relative to goal tracking. **(C)** Mean change in PCA index from training to test session for the paired (dark gray) and unpaired (light gray) groups. **(D–F)** Components of the PCA index. Average probability index **(D)**, bias index **(E)**, and latency index **(F)** over the last 3 days of training (dark blue) and in the test session (cyan) for the paired and unpaired groups. **(G,H)** Raw lever press count **(G)** and magazine entry count **(H)** over the last 3 days of training (dark blue) and in the test session (cyan) for the paired and unpaired groups. All panels, error bars indicate SEM. Double asterisk, *p* < 0.001; single asterisk, *p* < 0.05.

The next day, rats in both groups performed the PCA task in extinction (no rewards given) followed by a consumption test. As shown in Figure 2A, adolescent rats consumed many fewer sugar pellets following taste aversion conditioning (paired group; *Z* = 3.30, *p* < 0.001), but not following a sham devaluation procedure (unpaired group; *Z* = −1.60, *p* = 0.11). As we have previously shown in adults (Morrison et al., 2015), reward devaluation increased adolescents’ ratio of sign tracking to goal tracking: adolescents in the paired group, but not the unpaired group, had a significantly higher PCA index following reward devaluation (Figure 2B; paired: *Z* = −3.10, *p* = 0.002; unpaired: *Z* = −1.45, *p* = 0.15). Moreover, in a direct comparison, the PCA index of adolescents in the paired group trended higher than those in the unpaired group during the test session (*Z* = 1.60, *p* = 0.11). Although we would expect some increase in the PCA index even in the unpaired group due to the effects of extinction, the increase was significantly greater in the paired group (Figure 2C; *Z* = 1.98, *p* = 0.048).

Following reward devaluation, the paired group showed significant increases in the probability of lever deflection relative to magazine entry (Figure 2D; *Z* = −3.21, *p* = 0.001), bias towards lever deflection relative to magazine entry during the cue (Figure 2E; *Z* = −2.13, *p* = 0.03), and latency to magazine entry relative to lever deflection (Figure 2F; *Z* = −3.36, *p* < 0.001), whereas the unpaired group showed no such changes (all comparisons, *p* > 0.2). Moreover, two of the three indices were significantly or trending higher for the paired group than for the unpaired group during the test session (latency index, *Z* = 1.98, *p* = 0.048; probability index, *Z* = 1.70, *p* = 0.073; bias index, *p* > 0.2). Finally, for all measures except bias index, the change was significantly greater for the paired group than the unpaired group (data not shown; probability index, *Z* = 2.34, *p* = 0.02; latency index, *Z* = 2.58, *p* = 0.01). These changes were driven by both an increase in the average number of lever presses during the cue (Figure 2G; *Z* = −2.20, *p* = 0.03) and a robust decrease in the average number of magazine entries (Figure 2H; *Z* = 3.20, *p* = 0.001) among the paired group. Thus, reward devaluation resulted in an increase in the intensity of sign tracking behavior and a decrease in the intensity of goal-tracking behavior in adolescent rats.

**Figure 3:**
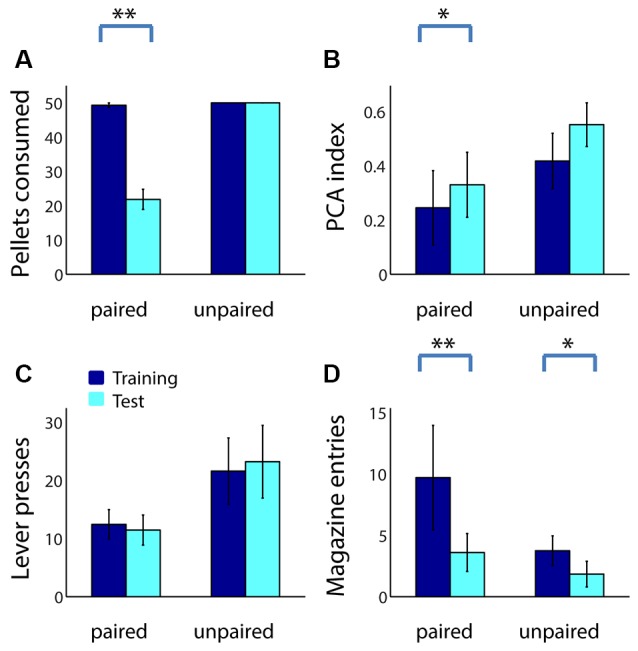
Reward devaluation decreases goal tracking, but not sign tracking, in adult rats. **(A)** The average number of sugar pellets consumed before (dark blue) and after (cyan) reward devaluation (paired group) or sham devaluation (unpaired group). **(B)** Average PCA index over the last 3 days of training (dark blue) and in the test session (cyan) for the paired and unpaired groups. Higher PCA index indicates more sign tracking relative to goal tracking. **(C,D)** Raw lever press counts **(C)** and magazine entry counts **(D)** over the last 3 days of training (dark blue) and in the test session (cyan) for the paired and unpaired groups. All panels, error bars indicate SEM. Double asterisk, *p* < 0.001; single asterisk, *p* < 0.05.

For comparison, we performed an identical reward devaluation experiment using 39 adult rats (20 in the paired group, 19 unpaired). Similar to adolescents, adults consumed fewer sugar pellets following taste aversion conditioning (Figure 3A; *Z* = 3.82, *p* < 0.001), but not following sham devaluation (*p* = 1). Devaluation effects were similar but weaker in adults compared to adolescents, as might be expected given their greater degree of sign tracking relative to goal tracking: the PCA index of adults in the paired group trended higher following devaluation (Figure 3B; *Z* = −1.79, *p* = 0.073), whereas the PCA index of adults in the unpaired group did not (*Z* = −1.49, *p* = 0.14). This change was primarily driven by a decrease in magazine entries among adults in the paired group (Figure 3D; *p* = 0.002), which was larger than the small but significant decrease in magazine entries in the unpaired group (*p* = 0.038) likely caused by extinction effects. Neither group showed a significant change in the number of lever presses (Figure 3C; *p* > 0.2).

### Individual Differences in Behavior and Devaluation Effects

**Figure 4:**
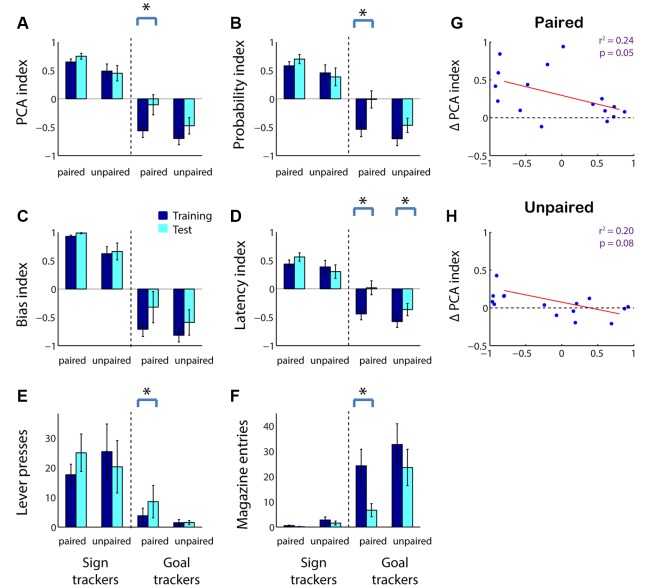
In adolescents, reward devaluation primarily causes a reduction in goal tracking among goal tracker individuals.** (A–D)** Average PCA index **(A)**, probability index **(B)**, bias index **(C)**, and latency index **(D)** among sign trackers (left-hand side of panels) and goal trackers (right-hand side of panels) before and after reward devaluation (paired group) or sham devaluation (unpaired group). Higher PCA index indicates more sign tracking relative to goal tracking. Dark blue, average over the last 3 days of training. Cyan, test session (in extinction). **(E,F)** Raw lever press count **(E)** and magazine entry count **(F)** among sign trackers (left-hand side of panels) and goal trackers (right-hand side of panels) before and after reward devaluation (paired group) or sham devaluation (unpaired group). Dark blue, average over the last 3 days of training. Cyan, test session. All panels, error bars indicate SEM. Asterisk, *p* < 0.05. **(G,H)** Average PCA index over the last 3 days of training plotted against change in PCA index for the paired **(G)** and unpaired **(H)** groups. Regression lines in red.

We next asked whether, in adolescents as in adults, sensitivity to reward devaluation is a characteristic that varies with an individual’s tendency to ascribe incentive salience to a cue. As shown in Figure 1, we divided subjects into sign trackers and goal trackers based on average PCA index: subjects with a PCA index >0 were categorized as sign trackers, and subjects with a PCA index <0 were categorized as goal trackers. We found that the population effects of reward devaluation (Figure 2) were almost entirely attributable to goal trackers. Only among goal trackers did PCA index show a significant increase following reward devaluation (Figure 4A; *Z* = −2.43, *p* = 0.015); the same was not true for sign trackers or following sham devaluation (all comparisons, *p* > 0.1). Furthermore, the PCA index of goal trackers in the paired group trended higher than goal trackers in the unpaired group during the test session (*p* = 0.11). Likewise, goal trackers, but not sign trackers, showed a significant increase in the probability of lever deflection relative to magazine entry (Figure 4B; *Z* = −2.67, *p* = 0.008) and a significant increase in latency to magazine entry relative to lever deflection (Figure 4D; *Z* = −2.67, *p* = 0.008) following reward devaluation (although there was no significant change in bias index; Figure 4C). Direct comparisons revealed that both of these indices were higher in the paired group than the unpaired group during the test session (probability index, *p* = 0.032; latency index, *p* = 0.024).

Raw behavior counts showed that the changes in PCA index and its components were predominantly due to a robust devaluation-induced decrease in magazine entries during the cue among goal trackers (Figure 4F; *Z* = 2.67, *p* = 0.008) accompanied by a small but significant increase in lever presses (Figure 4E; *Z* = −1.99, *p* = 0.046). In contrast, there was no significant change in behavior among sign trackers or following sham devaluation (all comparisons, *p* > 0.05).

Finally, we investigated whether the effects of devaluation can be predicted by an individual’s tendency towards sign-tracking or goal-tracking behavior on a subject-by-subject basis. Indeed, there was a significant negative correlation between an individual’s pre-devaluation PCA index and subsequent change in the PCA index (Figure 4G; *r*^2^ = 0.24, *p* = 0.05). A smaller negative relationship was seen in the unpaired group, likely due to the effects of extinction (Figure 4H; *r*^2^ = 0.20, *p* = 0.08). Thus, adolescents’ behavior is sensitive to reward devaluation to a degree commensurate with their baseline levels of goal tracking, whereas sign tracking is either unaffected or increased by reward devaluation.

## Discussion

In adult rats, sign-tracking behavior, compared with goal-tracking behavior, is relatively insensitive to reward devaluation, whether accomplished *via* pre-feeding (Patitucci et al., 2016; Conrad and Papini, 2018) or conditioned taste aversion (CTA; Morrison et al., 2015; Smedley and Smith, 2018; although devaluation can affect the approach to the cue under some circumstances: see Cleland and Davey, 1982; Derman et al., 2018). Because resistance to reward devaluation is one of the defining features of habitual actions (Balleine and O’Doherty, 2010), these findings imply that sign tracking is a habit-like behavior, although it is distinct from a classically defined habit because it arises from Pavlovian rather than instrumental contingencies (Dayan and Berridge, 2014).

In the current study, we used reward devaluation to determine whether the Pavlovian conditioned behavior of adolescent rats is more goal-driven or habit-like. As in previous studies (Anderson and Spear, 2011; Doremus-Fitzwater and Spear, 2011), we found that adolescents exhibit less sign-tracking behavior (lever deflections) than a comparable group of adults under normal, low-stress circumstances. We extend these findings to show that adolescents also engage in more apparent goal-tracking behavior (magazine entries) than adults. There are two possible explanations for this observation: on one hand, the increase in magazine entries might reflect a tendency by adolescents to place incentive salience on a more reward-proximal cue: the food magazine itself. On the other hand, adolescents might simply engage in more goal-directed behavior, and less habit-like behavior, than adults.

Our results support the latter hypothesis: just as in adults, the magazine-oriented behavior of adolescents is markedly sensitive to reward devaluation and therefore fits the definition of goal-directed behavior. When we subjected adolescent rats to CTA, in which they learned to associate sugar pellets with LiCl-induced illness, they dramatically reduced their interactions with the food magazine during the cue. This finding challenges the idea that CTA might be relatively ineffective in adolescents (Hammerslag and Gulley, 2014) because they are less sensitive to the aversive consequences of rewarding stimuli (Doremus-Fitzwater and Spear, 2016). Perhaps more importantly, it shows that adolescents’ magazine-directed behavior can fairly be termed “goal tracking,” rather than sign tracking directed towards the food magazine.

The current findings provide evidence against the hypothesis that adolescents are less goal-oriented and more “stimulus-driven” than adults (Ernst et al., 2011; Hammerslag and Gulley, 2014), even though there is evidence that adolescents are more responsive to reward-associated cues in certain contexts. Adolescents are faster to acquire cued behaviors associated with drug rewards (Schramm-Sapyta et al., 2011), slower to extinguish such behaviors (Anker and Carroll, 2010; Meyer and Bucci, 2016), and exhibit stronger cue-induced reinstatement of drug-seeking (Brenhouse and Andersen, 2008). Nevertheless, adolescent rats are no more likely than adults to transfer incentive salience to a cue; on the contrary, based on their relatively low levels of sign tracking, it seems that adolescents are considerably less likely than adults to ascribe independent motivational value to cues. Adolescents’ responsiveness to reward-associated cues might be better explained by their generally enhanced sensitivity to the rewarding properties of food and drugs (Friemel et al., 2010; Doremus-Fitzwater and Spear, 2016).

Indeed, there is a small but growing body of literature supporting the notion that adolescents can be more goal-driven than adults under the right circumstances. In addition to the finding that adolescents are less prone to sign tracking, studies have suggested that adolescents are less susceptible to habit formation (Serlin and Torregrossa, 2015) and that their cognition and behavior is more flexible compared to adults (Sturman and Moghaddam, 2012; Simon et al., 2013; Westbrook et al., 2018). This is consistent with adolescence as a time of enhanced exploratory behavior, novelty-seeking, and risk-taking (Douglas et al., 2003): adolescents are still learning the rules of their environment, including which stimuli and actions are most often followed by reward, and habit formation would be a hindrance to exploration and learning. Adults, on the other hand, can take advantage of the computational efficiency of engaging in cue-driven and/or habitual behavior.

The current findings complement the observation that adolescents perform more sign tracking, equivalent to or surpassing adult levels, when exposed to stressors such as social isolation combined with food restriction (Anderson et al., 2013; DeAngeli et al., 2017). Interestingly, food restriction by itself, as in the current study and that of Anderson et al. (2013), is inadequate to elevate adolescents’ sign tracking above that of adults; it may be the case that the stresses of food restriction and social isolation are additive, or that social isolation has an especially stressful impact on adolescent individuals. Overall, these studies imply that adolescents are fully capable of transferring incentive salience to cues, consistent with their adult-like expression of Pavlovian-instrumental transfer (Naneix et al., 2012), but tend to do so only when under stress, possibly as an adaptation to limit exploration when resources or social support are scarce. Indeed, there is evidence that the mesolimbic dopamine system, which is thought to underlie sign tracking (Flagel et al., 2011), is specifically vulnerable to stress-induced abnormalities in adolescents (Buwalda et al., 2011).

A substantial body of literature supports the notion that sign tracking and goal tracking are the behavioral outputs of two parallel reinforcement learning processes—akin to model-free and model-based learning, respectively (Clark et al., 2012; Huys et al., 2014). Although there has been limited investigation of model-free vs. model-based learning in adolescents, especially in animal models, the predominant view is that model-free learning is present throughout the lifespan, whereas model-based learning emerges slowly and is not fully integrated into behavior until adulthood (Decker et al., 2016; Potter et al., 2017). Our finding that adolescent rats perform more goal tracking and less sign tracking—and that such goal tracking is truly goal-oriented behavior, based on its sensitivity to reward devaluation—complicates this view.

There are a number of possible reasons for these differing results, including species differences between the development of these systems in humans vs. rodents. Although there is evidence that humans, including children, display sign tracking- and goal tracking-like behavior in a Pavlovian conditioning context (Garofalo and di Pellegrino, 2015; Joyner et al., 2018), it is currently unknown whether human adolescents exhibit less sign tracking and more goal tracking than adults. Moreover, there may be important developmental differences in the engagement of different learning systems during Pavlovian conditioning vs. instrumental tasks. In Pavlovian conditioning, the subject’s actions have no impact on reward delivery; in contrast, in instrumental tasks, reward delivery is contingent upon the performance of a certain action or actions. There is a large body of literature demonstrating that Pavlovian and instrumental conditioning engage distinct, though overlapping, brain circuits, and that these circuits can interact in complex ways to generate behavior (Dayan and Berridge, 2014).

The distinction between Pavlovian and instrumental contexts may also be a factor in the influence of extinction on adolescent behavior. In the current study, the test session was performed in extinction in order to minimize new cue-outcome learning. Prior studies have shown that sign tracking, compared to goal tracking, is resistant to extinction behaviorally (Ahrens et al., 2016) and in the context of cue-related neural activity (Gillis and Morrison, 2019). Compared with our prior report in adults, in adolescents, we see a larger apparent effect of sham devaluation (e.g., see Figure 4H). However, when we minimized the effect of extinction by analyzing a smaller number of trials at the beginning of the test session (5 instead of 10), the near-significant effects disappeared in the unpaired (sham) group, but not the paired group (data not shown). The relatively large effect of extinction, separate from reinforcer devaluation, might be accounted for by the larger number of goal trackers among the adolescent population; but it raises the intriguing possibility that extinction has a stronger effect on goal tracking in adolescents compared with adults. This would be consistent with the evidence that adolescents are more behaviorally flexible and sensitive to context and/or changing contingencies (Simon et al., 2013; Serlin and Torregrossa, 2015). On the other hand, several studies have found that adolescents, compared with adults, are more likely to exhibit perseverative behavior during extinction of Pavlovian and instrumental tasks (Sturman et al., 2010; Andrzejewski et al., 2011; Meyer and Bucci, 2016). Further experiments will be needed to clarify the factors—e.g., Pavlovian vs. instrumental setting, stress levels, length of training—that lead to greater or lesser sensitivity to extinction in adolescents.

Overall, the current study provides new evidence that, depending on task context and environment, compared with adults, adolescent behavior is neither disproportionately cue-driven, nor habit-like, nor the result of model-free learning processes. Although there is substantial evidence that the neural circuits associated with model-free learning develop earlier than circuits associated with model-based learning (Naneix et al., 2012), it is clear that simple forms of goal-directed behavior are fully operational in adolescence (and perhaps earlier) and are favored over the formation of habit-like Pavlovian behavior. In the future, a fuller understanding of adolescent behavioral processes, and how they differ from those of adults, is needed in order to improve prevention and treatment of substance use disorders, for which adolescents are at greater risk.

## Data Availability Statement

The datasets generated for this study are available on request to the corresponding author.

## Ethics Statement

The animal study was reviewed and approved by the University of Pittsburgh IACUC.

## Author Contributions

SM and BM designed the experiment. AR and SM carried out the experiment. SM analyzed the data and wrote the article with input from BM.

## Conflict of Interest

The authors declare that the research was conducted in the absence of any commercial or financial relationships that could be construed as a potential conflict of interest.
